# Role of c-Abl and nephrin in podocyte cytoskeletal remodeling induced by angiotensin II

**DOI:** 10.1038/s41419-017-0225-y

**Published:** 2018-02-07

**Authors:** Yiqiong Ma, Qian Yang, Zhentong Zhong, Wei Liang, Lu Zhang, Yingjie Yang, Guohua Ding

**Affiliations:** Division of Nephrology, Renmin Hospital of Wuhan University, Wuhan, 430060 China

## Abstract

Our previous study showed that angiotensin II (Ang II) exposure diminished the interaction between nephrin and c-Abl, then c-Abl mediated SHIP2-Akt pathway in the process of podocyte injury in vivo and vitro. However, the relationship between nephrin and c-Abl was unknown. Recently, various studies showed that nephrin was required for cytoskeletal remodeling in glomerular podocytes. But its specific mechanisms remain incompletely understood. As a nonreceptor tyrosine kinase involved in cytoskeletal regulation, c-Abl may be a candidate of signaling proteins interacting with Src homology 2/3 (SH2/SH3) domains of nephrin. Therefore, it is proposed that c-Abl contributes to nephrin-dependent cytoskeletal remodeling of podocytes. Herein, we observed that nephrin-c-Abl colocalization were suppressed in glomeruli of patients with proteinuria. Next, CD16/7-nephrin and c-Abl vectors were constructed to investigate the nephrin-c-Abl signaling pathway in podocyte actin-cytoskeletal remodeling. The disorganized cytoskeleton stimulated by cytochalasin D in COS7 cells was dramatically restored by co-transfection with phosphorylated CD16/7-nephrin and c-Abl full-length constructs. Further, co-immunoprecipitation showed that phosphorylated CD16/7-nephrin interacted with wild-type c-Abl, but not with SH2/SH3-defective c-Abl. These findings suggest that phosphorylated nephrin is able to recruit c-Abl in a SH2/SH3-dependent manner and detached c-Abl from dephosphorylated nephrin contributes to cytoskeletal remodeling in podocytes.

## Introduction

Podocytes, which play a key role in maintaining the kidney blood filtration barrier, are unique epithelial cells with a large cell body and long processes^1–3^. Microtubules and intermediate filaments form the framework of the podocyte cell body and primary processes, whereas the secondary processes are rich in actin^4^. The complex and unique actin cytoskeleton structure enables podocyte to adapt to fluctuating pressures and potentially harmful molecules contained in the primary filtrate^5–7^. Accordingly, remodeling of the actin cytoskeleton is closely related to foot process effacement, podocyte loss, and proteinuria^1^.

The foot processes of neighboring podocytes interdigitate and form a specialized intercellular junction called the slit diaphragm. During the initial phase of foot process effacement, the physiologic distribution of slit diaphragms is altered^8^. So, there is a direct relationship between the slit diaphragm and healthy podocyte function. The transmembrane protein nephrin, a member of the immunoglobulin superfamily, is expressed at the intercellular junction of differentiated podocytes and is of utmost importance in maintaining the integrity of the slit diaphragm^9,10^. Mutations in the gene coding for nephrin lead to congenital steroid-resistant nephrotic syndrome (NS) of the Finnish type, which is characterized by foot process dysmorphogenesis and a lack of intercellular junction formation and is associated with very severe proteinuria at birth^11^. Accumulating evidence suggests that nephrin prides a structural contribution to the filtration barrier and can also mediate signal transduction from the slit diaphragm in podocytes. The complex interaction among foot process morphology, slit diaphragm structure and actin remodeling are achieved through cell signaling events, in which nephrin acts as the core scaffold in governing actin dynamics in a number of different ways^4^. Nephrin has a large extracellular section with eight immunoglobulin G (IgG)-like domains and a cytoplasmic domain with tyrosine phosphorylation sites^12^. These tyrosine residues are known to bind proteins containing Src homology 2 (SH2) and Src homology 3 (SH3) domain, such as Nck^13^. Phosphorylated nephrin is involved in the regulation of multiple intracellular signaling pathways that influence actin polymerization. Moreover, nephrin has been shown to associate with other proteins, including Arp2/3, nWASP, synaptopodin, ZO-1, IQGAP1, and CD2AP, to regulate actin dynamics^13–15^. However, the mechanisms by which phosphorylated nephrin affects podocyte cytoskeletal remodeling remain unknown.

c-Abl is a ubiquitously expressed nonreceptor protein tyrosine kinase that binds diverse signaling molecules and exhibits distinct cellular functions depending upon its functional domains^16^. The SH2 and SH3 domains of c-Abl are considered as vital interaction and binding sites for various adaptor proteins^17^. Activated c-Abl is involved in an array of cellular processes, including immunity, infection, cytoskeletal remodeling, and apoptosis^18,19^. Our previous study demonstrated that c-Abl connects nephrin and the SHIP2-Akt pathway, which mediates the event of angiotensin II (Ang II)-induced podocytes injury. However, the relationship between nephrin and c-Abl has not been elucidated^20^. Here, we explore the role of the nephrin–c-Abl signaling pathway in podocyte cytoskeletal remodeling.

## Results

### Renal nephrin and c-Abl expression in NS patients

**Fig. 1:**
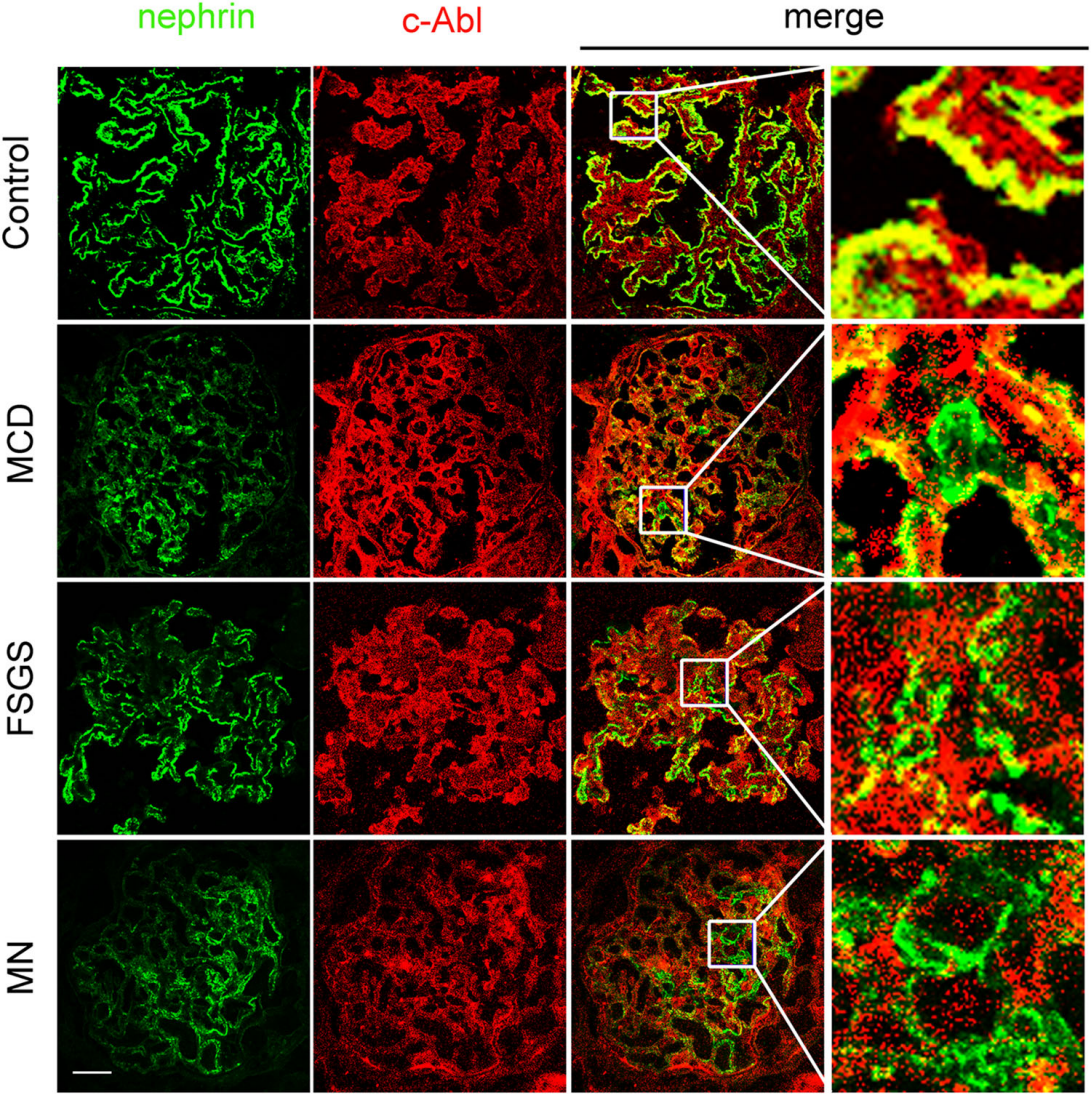
The expression of nephrin and c-Abl in glomeruli of participants. Double immunofluorescence staining of glomerular nephrin and c-Abl in the different groups (original magnification, ×400). Scale bar = 20 μm. MCD minimal change disease, FSGS focal segmental glomerulosclerosis, MN membranous nephropathy

Twenty-six patients with severe proteinuria, including 7 minimal change disease (MCD) patients, 8 focal segmental glomerulosclerosis (FSGS) patients, and 11 membranous nephropathy (MN) patients, were enrolled in this study, and renal biopsies were performed on all the patients. Four normal (control) kidney tissues were obtained from patients undergoing nephrectomy for renal neoplasm. No differences were observed between groups regarding age, gender, or blood pressure (BP) values; however, urinary albumin and serum creatinine values were higher in the experimental groups than in the control group (Table 1). As shown in Fig. 1, confocal microscopy revealed that nephrin-c-Abl colocalization was suppressed in glomeruli of experimental patients compared with control patients. These findings were consistent with our previous studies, which indicated that nephrin-c-Abl colocalization markedly decreased after podocyte injury in animals models and cultured cells^20^.

**Table 1:** Clinical features of each group individuals

	Control (*n* = 4)	MCD (*n* = 7)	FSGS (*n* = 8)	MN (*n* = 11)
Age (years)	52.5 ± 8.04	38.57 ± 11.43	46.25 ± 9.85	46.36 ± 7.18
Gender (male/femal)	2/2	4/3	5/3	6/5
SBP (mmHg)	122.5 ± 10.97	142.71 ± 12.23^*^	149.50 ± 7.09^*^	150.55 ± 10.05^**#**^
DBP (mmHg)	78.25 ± 5.37	94.14 ± 8.59^*^	91.25 ± 4.98^*^	92.54 ± 8.34^*^
Urinary albumin (g/24 h)	0.11 ± 0.03	3.57 ± 0.63^#^	3.9 ± 0.92^#^	4.04 ± 0.59^#^
Serum creatinine (μmol/L)	61.2 ± 7.37	89.74 ± 22.21^*^	102.85 ± 12.64^#^	93.23 ± 11.43^#^

*MCD* minimal change disease, *FSGS* focal segmental glomerulosclerosis, *MN* membranous nephropathy

*p < 0.05 compared with control group; #*p* < 0.01 compared with control group

### Effect of nephrin on the nephrin-c-Abl interaction in vitro

**Fig. 2:**
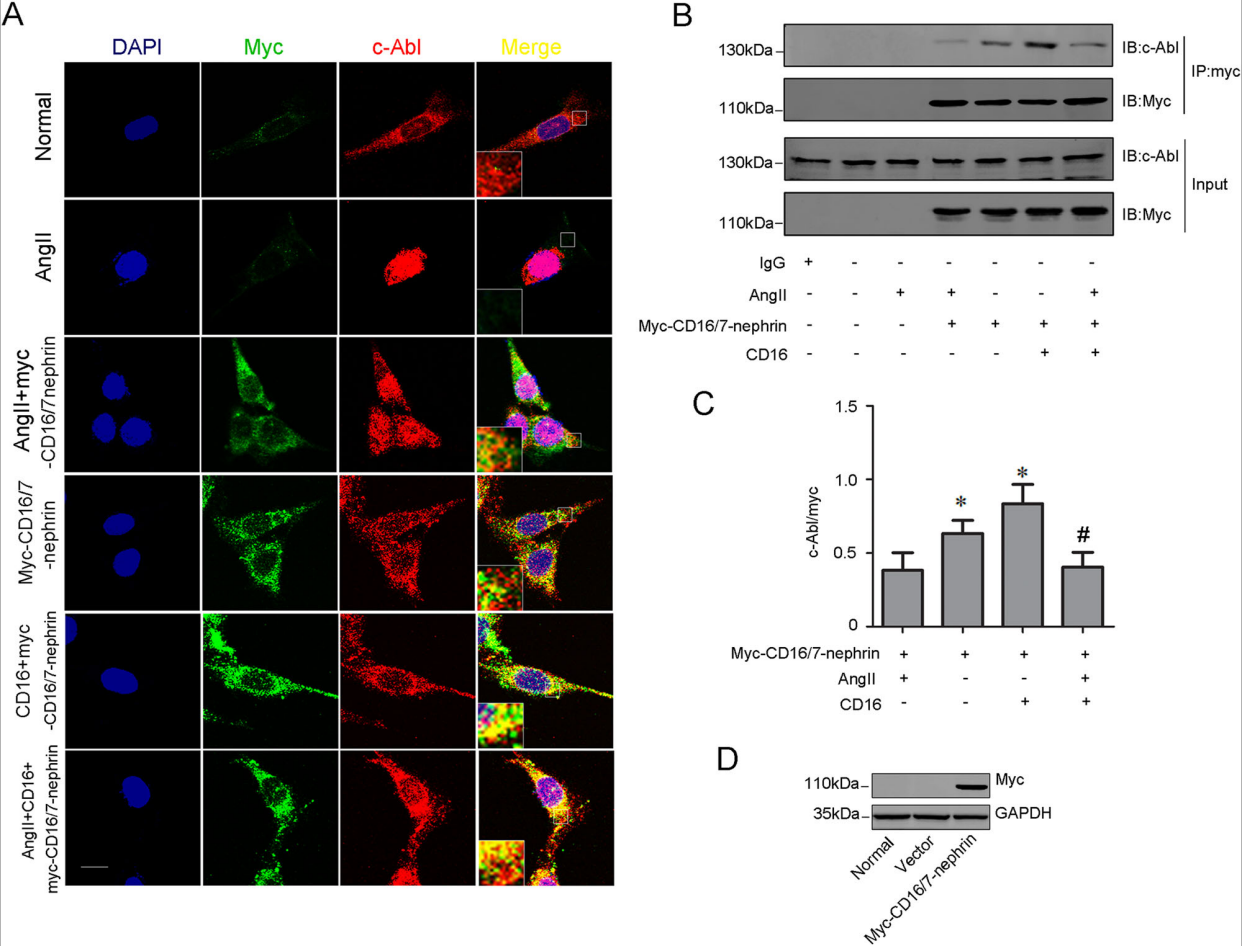
Phosphorylated nephrin recruites c-Abl in cultured podocytes. Podocytes were transfected with myc-CD16/7-nephrin constructs and then stimulated with Ang II or CD16 antibody. **a** Double immunofluorescence staining of myc and c-Abl in different groups (original magnification, ×400). Scale bar = 10 μm. **b**, **c** Representative co-immunoprecipitation results of the interaction between myc and c-Abl in podocytes (*n* = 6). *p < 0.05 compared with Ang II-induced podocytes transfected with myc-CD16/7-nephrin constructs; ^#^*p* < 0.05 compared with the CD16 antibody-pretreated podocytes transfected with myc-CD16/7-nephrin constructs. **d** Representative western blot of myc expression in podocytes transfected with vectors

Total and phosphorylated nephrin levels were significantly decreased in podocytes after Ang II stimulation; however, c-Abl small interfering RNA (siRNA) and STI571 (c-Abl inhibitor) pretreatment had no influence on total nephrin and phosphorylated nephrin levels^20^. To further investigate the nephrin-c-Abl signaling pathway, the myc-CD16/7-nephrin construct was transfected into podocytes and nephrin phosphorylation was induced following clustering with the CD16 antibody, as reported previously^13,21,22^. As shown in Fig. 2a, the myc-CD16/7-nephrin chimera failed to colocalize with endogenous c-Abl. CD16 antibody induction enhanced the colocalization of the myc-CD16/7-nephrin chimera and c-Abl. The same results were obtained in the co-immunoprecipitation assay (Fig. 2b, c). Thus, the phosphorylated nephrin was available to colocalize with c-Abl, and nephrin-c-Abl signal transduction depends on nephrin phosphorylation/dephosphorylation.

### Role of phospho-nephrin in Ang II-induced podocyte cytoskeletal reorganization

**Fig. 3:**
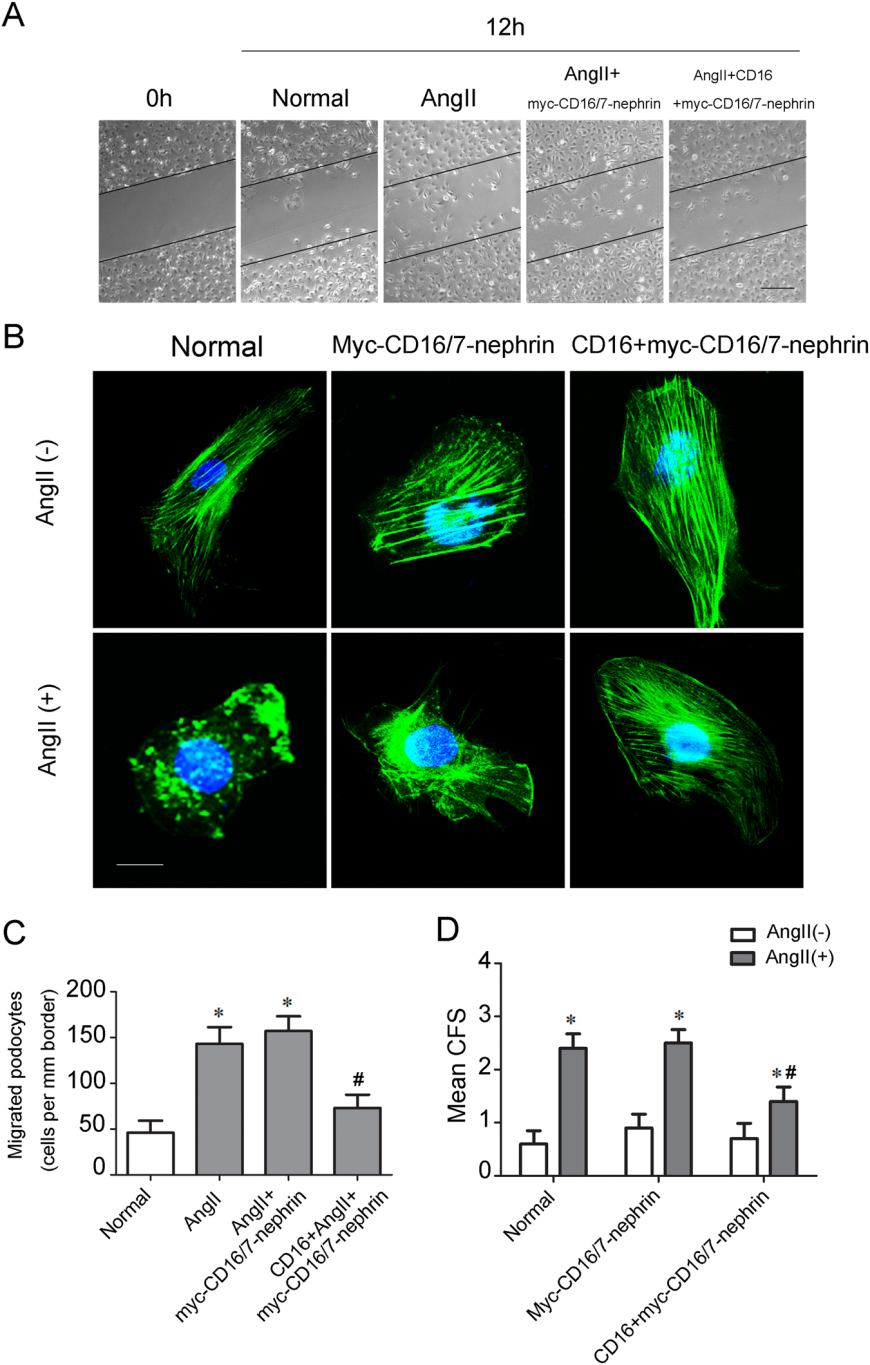
Nephrin phosphorylation restores Ang II-induced podocyte cytoskeleton reorganization. Podocytes were transfected with myc-CD16/7-nephrin constructs and then stimulated with Ang II or the CD16 antibody. **a**, **c** Representative migration results and quantification of different podocytes groups (*n* = 6). Scale bar = 100 μm. *p < 0.05 compared with control podocytes. ^#^*p* < 0.05 compared with the Ang II-induced podocytes transfected with myc-CD16/7-nephrin constructs. **b**, **d** FITC-phalloidin staining and quantification of the cortical F-actin score for each group of differentiated podocytes (*n* = 6). Scale bar = 10 μm. *p < 0.05 compared with the podocytes treated without Ang II in each group. ^#^*p* < 0.05 compared with the Ang II-induced podocytes transfected with myc-CD16/7-nephrin constructs

Our previous results showed that the phosphorylation of nephrin is necessary for c-Abl recruitment. In this study, we used the myc-CD16/7-nephrin construct to investigate the role of phospho-nephrin in Ang II-induced podocyte cytoskeletal reorganization. As shown in Fig. 3a, c, transfection of the myc-CD16/7-nephrin chimera without CD16 antibody induction did not alter podocyte migration capability. Addition of the CD16 antibody to the media inhibited cell migration after Ang II stimulation. Stress fibers were disorganized in Ang II-treated podocytes with or without transfection (Fig. 3b, d). Ang II-induced cytoskeletal disorganization was restored in the presence of the CD16 antibody (Fig. 3b, d). These findings indicate that nephrin phosphorylation alleviated podocyte cytoskeletal reorganization induced by Ang II. Notably, we confirmed that phosphorylated nephrin was available to colocalize with c-Abl, suggesting that nephrin-c-Abl signal-transduction-mediated podocyte cytoskeletal reorganization.

### Involvement of the interaction between phospho-nephrin and c-Abl in cytoskeletal regulation

**Fig. 4:**
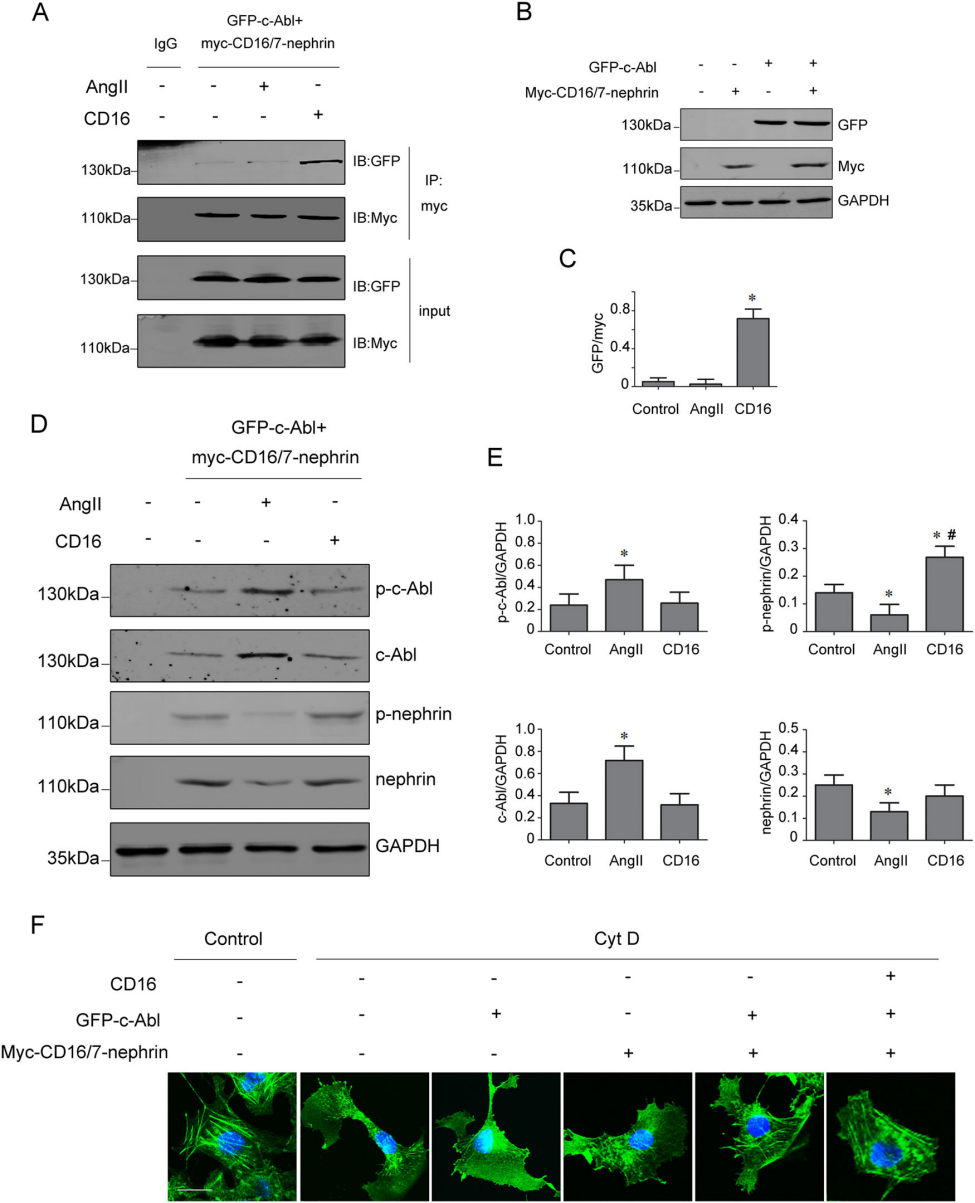
The interaction between phospho-nephrin and c-Abl in COS7 cells co-transfected with myc-CD16/7-nephrin and GFP-c-Abl. COS7 cells were co-transfected with myc-CD16/7-nephrin and GFP-c-Abl constructs and then stimulated with Ang II or the CD16 antibody. **a**, **c** Representative co-immunoprecipitation results of the interaction between myc and GFP in COS7 cells (*n* = 6). *p < 0.05 compared with control group. **b** Representative western blot of GFP and myc expression in COS7 cells transfected with vectors. **d**, **e** Representative western blot of nephrin, phospho-nephrin, c-Abl, and phospho-c-Abl levels in different COS7 cell groups (*n* = 6). *p < 0.05 compared with the control group. ^#^*p* < 0.05 compared with the Ang II group. **f** The FITC-phalloidin staining of the actin cytoskeleton in COS7 cells pretreated with Cyt D and transfected with plasmids (original magnification ×400). Scale bar = 10 μm. Control COS7 cells transfected with constructs, Ang II COS7 cells transfected with constructs and treated with Ang II, CD16 COS7 cells transfected with constructs and treated with the CD16 antibody, Cyt D cytochalasin D

To further validate the recruitment of c-Abl to phosphorylated nephrin, GFP-c-Abl plasmids were co-transfected with myc-CD16/7-nephrin constructs in COS7 cells (Fig. 4b). After Ang II stimulation, total and phosphorylated c-Abl levels were increased, but total and phosphorylated nephrin levels were decreased in COS7 cells (Fig. 4d, e). By contrast, CD16 antibody treatment induced nephrin phosphorylation (Fig. 4d, e). Moreover, myc-CD16/7-nephrin co-immunoprecipitation with GFP-c-Abl occurs in the presence of the CD16 antibody (Fig. 4a, c). The stress fibers in COS7 cells were obviously disrupted by cytochalasin D (Cyt D, a cytoskeleton depolymerizing agent). Transfection with constructs did not rescue the stress fibers of the F-actin cytoskeleton in Cyt D-treated COS7 cells, but CD16 antibody stimulation dramatically restored Cyt D-induced cytoskeletal disorganization (Fig. 4f).

### The SH3 and SH2 domains of c-Abl are phospho-nephrin binding sites

**Fig. 5:**
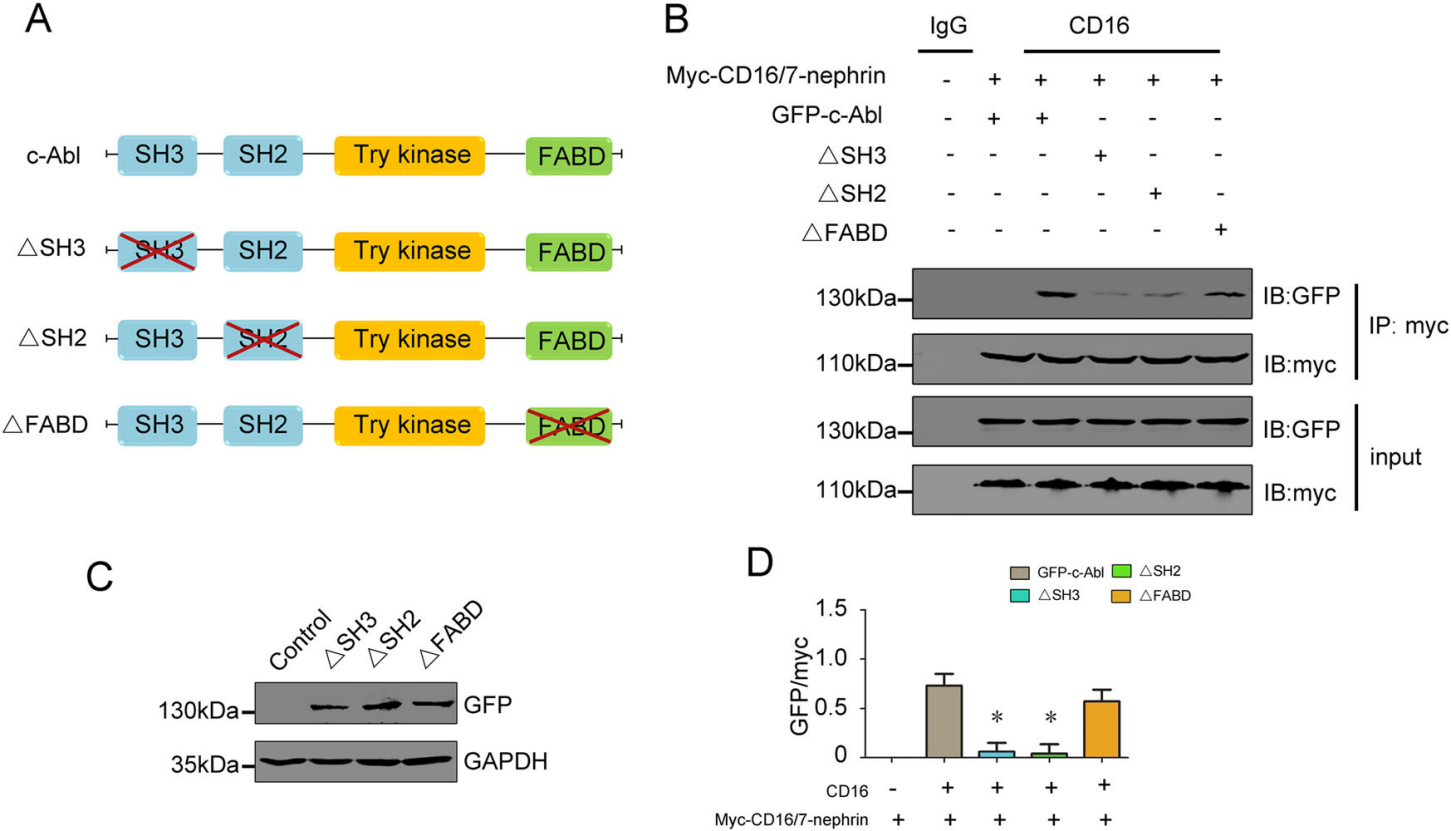
Phospho-nephrin interacts with c-Abl via SH2 and SH3 domains. **a** Schematic of c-Abl plasmids. **b**, **d** Representative co-immunoprecipitation results of the interaction between myc and GFP in COS7 cells (*n* = 6). *p < 0.05 compared with CD16 antibody-pretreated COS7 cells co-transfected with myc-CD16/7-nephrin and c-Abl full-length plasmids. **c** Representative western blot of GFP and myc expression in COS7 cells transfected with vectors. ΔSH2 c-Abl mutant plasmids with SH2 domain deletion, ΔSH3 c-Abl mutant plasmids with SH3 domain deletion, ΔFABD c-Abl mutant plasmids with FABD domain deletion

**Fig. 6:**
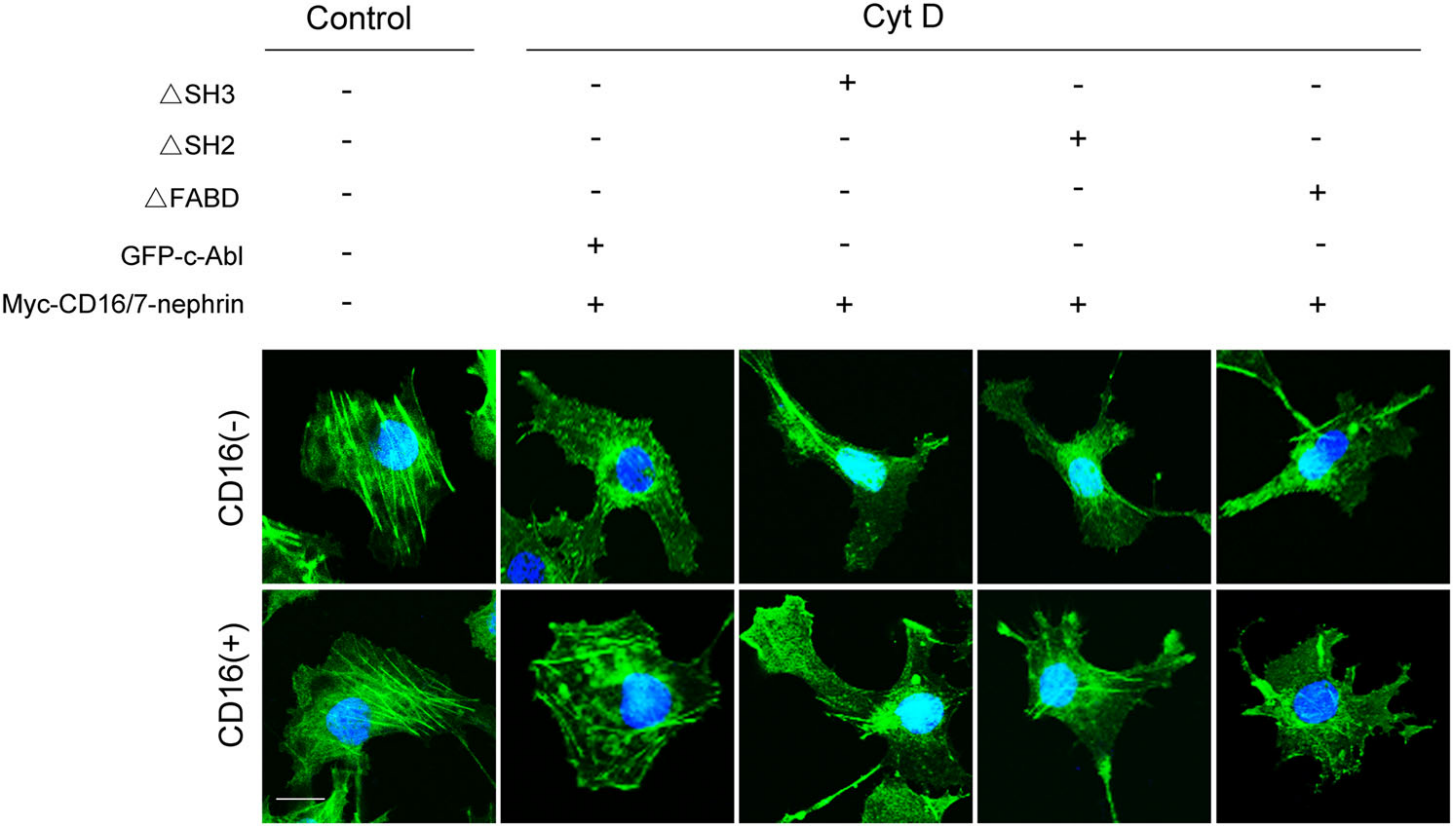
Involvement of the phospho-nephrin and c-Abl interaction in cytoskeletal regulation. The FITC-phalloidin staining of actin cytoskeleton in COS7 cells pretreated with Cyt D and transfected with c-Abl wild-type or mutated plasmids (original magnification, ×400). Scale bar = 10 μm

Lastly, we proceeded to identify the domains of c-Abl that are responsible for binding to nephrin. Wild-type or mutant c-Abl plasmids (ΔSH2, ΔSH3, and ΔFABD) were co-transfected with myc-CD16/7-nephrin constructs in COS7 cells (Fig. 5a, c). Co-immunoprecipitation analysis revealed that the SH2 and SH3 domains are necessary for the binding of c-Abl to phospho-nephrin (Fig. 5b, d). In addition, neither the ΔSH2 or ΔSH3 constructs rescued Cyt D-induced cytoskeletal disorganization (Fig. 6).

## Discussion

Actin remodeling, which mediates several biological activities, is a vital regulator of cell architecture and structure. It enables cells to adapt to environmental changes. The podocyte is the classical archetype of a terminally differentiated cell, with a rich actin cytoskeleton that underlies its flexibility under conditions of fluctuating pressure. Accumulating evidence has revealed that mutations in genes encoding proteins involved in maintaining of actin cytoskeleton stability in podocytes lead to glomerular disorders. Moreover, dysregulation of the actin cytoskeleton occurs following podocyte injury^23,24^. In this regard, it is meaningful to discuss the potential signaling pathways involved in the actin remodeling after podocyte injury.

Ang II, as the primary effector of the renin–angiotensin system, is known to participate in cellular pathological processes such as inflammation, apoptosis, and fibrosis by acting upon the Ang I-type receptor^25^. Recent studies have shown that Ang II is associated with kidney disease progression and podocyte injury^26,27^. Herein, we observed that Ang II induction led to stress fiber disorganization in normal podocytes, which was consistent with the findings of previous studies.

Nephrin is considered a vital signaling molecule in the slit diaphragm and regulates podocyte activity. Our previous study showed that the level of nephrin and phospho-nephrin expression obviously decreased when podocytes were injured^28^. In addition, nephrin phosphorylation is considered important for the maintenance of the podocyte health^14,29^. However, the mechanism is still unclear. To confirm the key role of phospho-nephrin, a CD16/7-nephrin chimera was used in certain experiments. The data showed that dephosphorylated nephrin failed to restore the disordered actin, whereas phosphorylated nephrin rescued the actin cytoskeletal remodeling. The intracellular domain of nephrin harbors potential binding sites for adaptor proteins, but the specific mechanisms of nephrin signal transduction and cytoskeleton regulation require further exploration.

Proteins containing SH2 or SH3 domains, such as Nck, podocin, and CD2AP, are thought to bind the cytoplasmic domain of nephrin^13–15^. Intriguingly, as a ubiquitously expressed nonreceptor protein tyrosine kinase, c-Abl participates in diverse cellular events and contains functional SH2 and SH3 domains^17^. Our previous studies demonstrated that c-Abl is a molecular chaperone of nephrin signaling^20,30^. After Ang II induction, c-Abl is released from nephrin and promotes the activation of the SHIP2-Akt signaling pathway, which contributes to podocyte injury.However, the specific mechanisms of interaction between nephrin and c-Abl remain unknown. Therefore, we hypothesize that c-Abl can interact with nephrin via its SH2 and SH3 domains and thus mediate actin cytoskeleton reorganization. The results showed that the expression of c-Abl increased and that it translocated from the cytoplasm to the nucleus in response to podocyte injury. Previous reports showed that c-Abl is present in two distinct configurations under different conditions: an inactivated orbicular configuration and an activated linear configuration when exposed to tyrosine kinases^31,32^. Furthermore, the SH2 and SH3 domains are known to be important for c-Abl autoinhibition^33^. We observed that c-Abl and nephrin were co-expressed in podocytes, and Ang II stimulation induced a greater reduction in nephrin-c-Abl colocalization. These data provide additional evidence that identifying c-Abl is a novel downstream effector of nephrin in the maintenance of podocyte cytoskeleton stability.

Our previous study demonstrated that nephrin phosphorylation levels and the c-Abl-SHIP2 interaction play key roles in maintaining podocyte health^20^. In this study, nephrin-c-Abl colocalization was markedly suppressed in glomeruli of patients with severe proteinuria, which indicated that nephrin-c-Abl signaling was involved in the process of podocyte injury. To further identify the relationship between nephrin and c-Abl, wild-type plasmids were co-transfected into COS7 cells. Unfortunately, we did not observe the colocalization of nephrin and c-Abl (data not shown). To further study the importance of nephrin phosphorylation, a CD16/7-nephrin chimaera was created as a model of nephrin phosphorylation Phosphorylated nephrin recruited c-Abl and reversed the disordered actin cytoskeleton. Accumulating evidence suggests that disruption of nephrin phosphorylation leads to podocyte injury and renal damage^34,35^. Together with these previous findings, our results indicate that the phosphorylation state of nephrin is crucial for its ability to interact with c-Abl and stabilize the actin cytoskeleton. Following podocyte injury, nephrin is dephosphorylated and releases c-Abl, which accelerated actin remodeling.

**Fig. 7:**
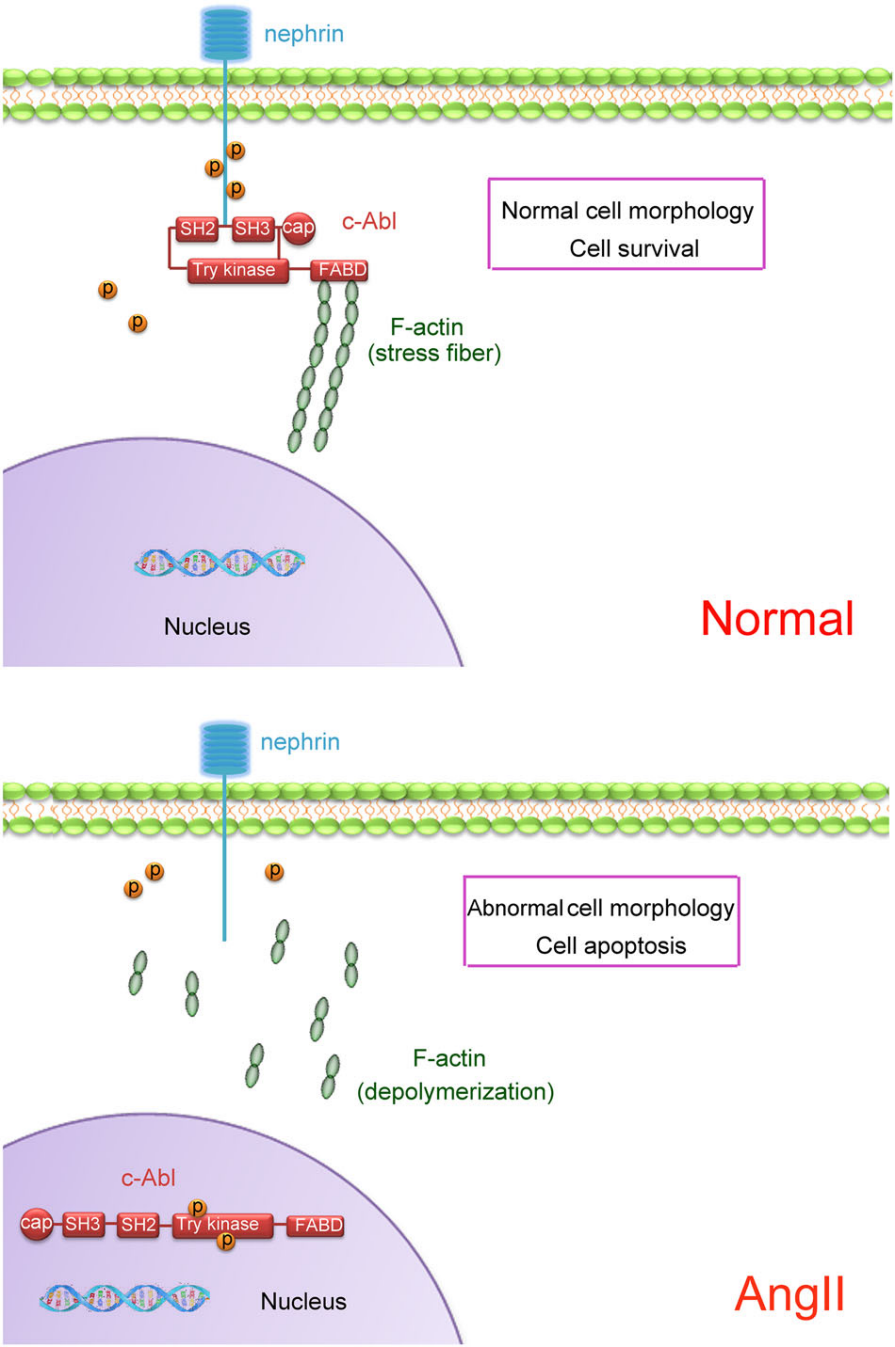
Schematic of the molecular model proposed in this study

The activation of c-Abl is associated with cellular events such as cell growth, proliferation, and migration^36,37^. The SH2 and SH3 domains of c-Abl are regarded as functional modules that interact with other proteins^33^, and the F-actin binding domain enables c-Abl to mediate actin remodeling^38^. Upon deletion of the SH2 or SH3 domain of c-Abl, phospho-nephrin failed to interact with c-Abl, and the actin disorder was not restored. These results verified that c-Abl interacts with phospho-nephrin via the SH2 and SH3 domains to regulate the actin cytoskeleton regulation. Taken together, our findings reveal that phospho-nephrin recruits c-Abl and that this complex maintains the podocytes cytoskeleton stability; furthermore, detachment of c-Abl and dephospho-nephrin lead to actin disruption after podocyte injury induced by Ang II (Fig. 7).

In closing, our observations suggest that c-Abl functions as a connector between the nephrin signaling hub and the cytoskeleton network in podocytes. We further extend the knowledge of the nephrin signaling pathway and the nephrin-c-Abl complex in regulating actin dynamics in podocytes. Thus, the findings presented herein further clarify the regulatory mechanisms of podocyte survival and provide new ideas and therapeutic strategies for addressing podocyte injury.

## Materials and methods

### Study population

Twenty-six eligible patients (aged 22–58 years) with NS, who were diagnosed in the Division of Nephrology, Renmin Hospital of Wuhan University, Wuhan, People's Republic of China, from May 2014 to September 2015 were selected; these patients had renal biopsy-proven MCD (7 patients), FSGS (8 patients), or MN (11 patients). Patients were excluded from the study if they had secondary NS. The clinical data are shown in Table 1. The control group comprised four patients with renal neoplasm, and normal kidney tissues were obtained from these patients by nephrectomy. The study protocol was approved by the Ethics Committee of Renmin Hospital of Wuhan University. All experiments were performed in accordance with the approved guidelines of Wuhan University. The research complied with the Declaration of Helsinki. Written informed consent was obtained from the patients for publication of this study and any accompanying images.

### Cell culture

Conditionally immortalized murine podocytes were kindly provided by Dr. Peter Mundel (Mount Sinai School of Medicine, New York, NY, USA) and were grown at 33 °C in RPMI-1640 medium (HyClone, USA) containing 10% heat-inactivated fetal calf serum (Gibco, USA), 100 U/ml penicillin G, 100 μg/ml streptomycin, and 10 U/ml recombinant mouse interferon-γ (Sigma, USA) in the presence of 5% CO_2_. Then, podocytes were cultured at 37 °C in interferon-γ-free medium for 7–14 days to induce differentiation and some cell groups were pretreated with STI571 (10 μM, 30 min, Enzo Life Sciences, Switzerland), which suppresses the phosphorylation of c-Abl.

COS7 cells were purchased from the Cell Resource Center of Shanghai Institutes for Biological Sciences, Chinese Academy of Sciences, and cultured in a 37 °C incubator in Dulbecco's modified Eagle's medium (HyClone, USA) containing 10% fetal calf serum (Gibco). When the cells reached approximately 60% confluence, 20 μg/ml Cyt D (Enzo Life Sciences) was added to the medium for 30 min to depolymerize the actin cytoskeleton.

### Plasmid construction and transfection

The full-length c-Abl (pCX-EGFP-c-Abl) expression plasmid was supplied by Addgene (plasmid #15007). ΔSH2, ΔSH3, and ΔFABD c-Abl mutant plasmids were constructed by deleting the SH2 domain, SH3 domain, or FABD domain, respectively. A construct encoding a fusion protein consisting of the CD16 extracellular domain, CD7 transmembrane domain, and nephrin cytoplasmic domain was generated using PCR-based techniques and tagged with myc (myc-CD16/7-nephrin).

COS7 cells were transfected with complexes containing the relevant plasmids and X-tremeGENE HP DNA Transfection Reagent (Roche, Germany) according to the protocol of the manufacturer.

### c-Abl siRNA transfection

Transfection of c-Abl siRNA was performed according to the HiPerFect Transfection Reagent Handbook (Qiagen, Germany). Briefly, 2 × 10^5^ cells were seeded in 6-well plates and transfected with complexes composed of 10 nM c-Abl siRNA or negative control scrambled siRNA and HiPerFect transfection reagent under normal growth conditions for 24 h.

### Western immunoblotting

Cells were lysed in RIPA buffer (150 mM sodium chloride, 1.0% Triton X-100, 0.5% sodium deoxycholate, 0.1% sodium dodecyl sulfate, and 50 mM Tris, pH 8.0) with protease/phosphatase inhibitors, and the lysates were centrifuged at 12,000 rpm for 20 min at 4 °C. Then, the protein samples were mixed with loading buffer and boiled at 95–100 °C for 5 min. The proteins were separated on 10% sodium dodecyl sulfate-polyacrylamide gels and then transferred to nitrocellulose membranes (GE Healthcare, Fairfield, CT, USA) by semidry blotting. Nephrin guinea pig polyclonal antibody, 1:500 (Progen Biotechnik, Heidelberg, Germany); p-nephrin(Y1217) rabbit monoclonal antibody, 1:1,000 (Epitomics, Burlingame, CA, USA); c-Abl rabbit polyclonal antibody, 1:500 (Cell Signaling Technology, Boston, MA, USA); p-c-Abl(Y412) rabbit polyclonal antibody, 1:500 (Cell Signaling Technology); Myc mouse monoclonal antibody, 1:2,000 (Medical and Biological Laboratories Co., Japan); GFP rabbit polyclonal antibody, 1:5,000 (Medical and Biological Laboratories Co.) and glyceraldehyde 3-phosphate dehydrogenase mouse monoclonal antibody, 1:2,000 (Antgene, Hubei, China) were used as primary antibodies. An Alexa Fluor 680/790-labeled goat anti-rabbit/goat anti-mouse IgG antibody (1:10,000; LI-COR Biosciences, Lincoln, NE, USA) was used as the secondary antibody, and the blots were visualized using a LI-COR Odyssey Infrared Imaging System.

### Immunofluorescence assay

Frozen kidney sections were blocked with 5% bovine serum albumin for 30 min at 37 °C and the cell-climbing film (cells growing on a glass slide) was fixed in 4% paraformaldehyde for 30 min at 4 °C. The sections were incubated with a mixture of primary antibodies (nephrin guinea pig polyclonal antibody, 1:50, Progen Biotechnik; c-Abl rabbit polyclonal antibody, 1:50, Cell Signaling Technology) overnight at 4 °C. Fluorescein isothiocyanate (FITC)/tetramethylrhodamine-conjugated IgG was used as secondary antibodies at 37 °C for 90 min in the dark. All microscopic images were recorded using a confocal microscope (Olympus, Japan).

### Co-immunoprecipitation

Co-immunoprecipitation experiments were performed using a kit (Beyotime, China) according to the manufacturer’s instructions. Briefly, total protein from cultured cells was extracted using lysis buffer (20 mM Tris, 150 mM NaCl, 1.0% Triton X-100, 5 mM EDTA, and 1 mM phenylmethylsulfonyl fluoride, pH 7.5). A c-Abl rabbit polyclonal antibody (1:200, Cell Signaling Technology) or myc mouse monoclonal antibody (5 μg/500 μg total protein, Medical and Biological Laboratories) was added to the protein samples, which were then rotated overnight at 4 °C. Then, the samples were mixed with 40 µl of protein A+G agarose beads and incubated for 3 h at 4 °C. The beads were mixed with 1 × Lane Marker Sample Buffer. After being boiled at 95–100 °C for 5 min, the samples were analyzed by western blotting.

### Staining of the actin cytoskeleton

The cell-climbing film (cell growing on a glass slide) was fixed in 4% paraformaldehyde for 30 min at 4 °C, then washed with ice-cold phosphate-buffered saline (PBS) three times for 5 min and incubated with FITC-phalloidin (2.5 μg/ml, Sigma-Aldrich) for 1 h at 37 °C. The nuclei were counterstained with 4′,6-diamidino-2-phenylindole (Antgene) for 5 min in the dark. All microscopic images were recorded using a confocal microscope (Olympus, Japan). The F-actin cytoskeletal reorganization of podocytes was assessed as previously described: 0, no cortical F-actin, normal stress fibers; 1, cortical F-actin deposits on less than half of the cell border; 2, cortical F-actin deposits on more than half of the cell border; and 3, complete cortical ring formation and/or total absence of central stress fiber^39^.

### Cell migration assay

Podocytes were prepared under control or experimental conditions in six-well culture plates. Two wounds were made to each well. The detached cells were subsequently removed by gently washing with PBS. The cells were then cultured at 37 °C for another 12 h. Images of the gaps were obtained 0 and 24 h after wounding with an inverted phase-contrast microscope. The number of cells crossing the 1-mm wound border was calculated. Three independent experiments were performed.

### Statistical analysis

The data are presented as the mean ± SEM, and the statistical analyses were performed using SPSS, version 17.0. Statistical comparisons of groups were conducted with one-way analysis of variance, and the least significant difference test was used for multiple comparison. The results were considered statistically significant at *p* < 0.05.
